# Medicine

**Published:** 1904-08

**Authors:** Nelson W. Wilson

**Affiliations:** Buffalo. N.Y.


### Medicine.

CONDUCTED BY NELSON W. WILSON, M. D., Buffalo, N.Y.

### TYPHOID PERFORATIONS.

FRANK, (*Jour. Amer. Med. Assoc.,* April 2, 1904,) in reporting a case of typhoid perforation discusses the literature and gives the following summary: Perforations are to be expected in about 2.5 per cent. of all cases of typhoid fever. Prompt surgical intervention is the best and only logical treatment. Early diagnosis is most desirable and will be the means of greatly reducing the mortality, as 55 to 60 per cent. would recover. Diagnosis sufficiently early to achieve these results can only be made by careful watching, treating all cases as serious ones and a proper interpretation of the early, even the preperforative symptoms, the suggestions of Cushing as to this stage being of value. At the first indication of this stage have the surgeons in consultation and be prepared to operate. The sphygmomanometer should come into general use as an important aid to diagnosis. More cases die from delay than error in surgical technic. Therefore, in doubtful cases, though mistake may be made and no perforation be found, operate. No case, unless dying, is so desperate as to be beyond some hope of saving. So in operating lose no time, and be sure to drain. Get into the abdomen quickly and get out more quickly. —*Therapeutic Review.*

---

### THE THERAPEUTIC USE OF BENZOIN.

ACCORDING to Colton (*Therapeutic Gazette,* 1903, Vol. XXVII., p. 441,) the therapeutic value of benzoin depends, chiefly, on the benzoic acid which it contains.

Benzoic acid is an efficient antiseptic and even germicide. According to Brunton, it is an antiseptic in the proportion of 1 part to 1,000, and begins its germicidal action in strength of 4 parts to 1,000. Colton ranks the tincture of benzoin above all other occlusive dressings for small wounds. It is very useful for the treatment of cracked nipples, bed sores, chapped hands and fissures of the anus, and the like. Chronic forms of eczema are benefited by painting them with a tincture, or dusting them with benzoate of bismuth.—*Therapeutic Review.*

---

VALUE OF SALINE INFUSION IN ENTERIC FEVER.

D. G. Marshall, (*Lancet*, 1903, Vol. II., p. 1152,) reports the case of a woman of 25, who developed typhoid fever eight weeks after the birth of a child. The attack was a very intense one, the temperature at a very early date remaining about 104. During this time cold packs kept the temperature in check. On the nineteenth day, with a temperature of 105 and very weak heart sounds, the patient had a chill. Ordinary heart stimulants did not rouse the patient, and 20 ounces of saline solution were injected. The patient answered to this very nicely. On the twentieth day another chill occurred and again the heart sounds became very weak; 25 ounces were injected. On the twenty-sixth day a hemorrhage necessitated the injection of more salt solution and 25 ounces were injected twice within an interval of 12 hours. Improvement continued until the forty-second day when a relapse began. On the fifty-second day another chill was noted, the temperature rising to 106.2, the pulse to 160; 30 ounces of saline solution were injected with considerable improvement.

The patient had another relapse some time later but ultimately recovered. The general treatment throughout was fresh chlorine water and other intestinal antiseptics, sulphuric acid and opium to restrain the diarrhea, and 20-grain doses of chloride of calcium in hemorrhage. The author is certain the patient's life was saved by the saline infusion.—*Therapeutic Review.*

---

TREATMENT OF HEMORRHAGE IN TYPHOID FEVER.

Moore, (*Practitioner*, 1904, Vol. LXXVII., p. 139,) believes a profound intestinal hemorrhage in typhoid fever is a less dangerous symptom than repeated small bleedings. A hemorrhage from the bowels in the third or fourth week is a much more dangerous symptom than that occurring in the earlier stages of the fever. For the purpose of controlling the hemorrhage he recommends, first, absolute rest, not only of the patient but also of the bowels; this is obtained by withholding food for several hours, and by the free exhibition of opium, preferably in the form of hypodermic injections of morphine. Ice poultice or ice bag is laid over the right side of the abdomen. He frequently employs the following formula:

| | | |
|---|---|---|
| ℞ | Acidi tannici | gr. x. |
| | Tincturæ opii | m. x. |
| | Spiritus terebinthinæ | m. xv. |
| | Mucilaginis acaciæ | ʒij. |
| | Tinct. chloroformi comp. | m. xx. |
| | Aquæ menthæ piperitæ ........ ad | ℥j. |

Sig.—Tablespoonful at a dose.

When the hemorrhage is so profuse as to threaten life, ice-water enematas or a hypodermic injection of salt solution may be employed; in such cases 20-grain doses of chloride of calcium every few hours are of value, while adrenalin or cornutin may also be employed.—*Therapeutic Review.*

---

### TUBERCULOUS PERICARDITIS, WITH A REPORT OF CASES.

STOCKTON, (*American Medicine,* June 11, 1904,) gives the clinic and pathologic manifestations of three cases of tuberculous pericarditis with the following summary:

1. Tuberculous pericarditis is not a rare affection.
2. The diagnosis is usually not made except in cases with simultaneously active tuberculous processes in other parts.
3. The concurrence of pleurisy with blood stained effusion may be regarded as suggestive.
4. The pericarditis may be of a chronic obliterative type, or there may be massive effusion, generally sanguinolent, but rarely purulent.
5. It may be acute, continuing for but a few days, or chronic, existing for many months.
6. It may be a part of a multiple serositis, and the proportion of cases in which at least one or more of the pleural cavities are involved is remarkable.
7. The disease is to be regarded as a secondary affection, although from a clinical standpoint of view, some cases may be looked upon as primary.
8. The point of origin of the infection is often found in the bronchial and mediastinal lymph-nodes, although these may be quite exempt from the disease. The infection may be direct from continuity of tuberculous tissue, or by transmission through lymph vessels, or through the circulation.

9. The heart may be greatly enlarged, or normal in size, or even somewhat small.

10. Some observers believe that occasionally the process subsides and that comparative cure results.—*Medical Fortnightly.*

---

### REMARKS ON FICKER'S TYPHOID DIAGNOSTICUM.

EHRSAM, (*Meunchener Med. Wochenschrift*, No. 15, 1904.)—The publication of Dr. Meyer (*Berlin klin. Wochenschrift*, No. 9, 1904,) on the reliability of Ficker's reagent inspired the author to further investigation. The writer's experiments were carried out in the laboratory of Prof. Leubuscher, in Meiningen. In brief, Ehrsam's results are the following:

1. A carpenter, 42 years old, with a diagnosis of typhoid fever showed a positive reaction with Ficker's reagent in the third week and during convalescence.
2. A woman, 36 years; typhoid; reaction positive in the latter part of the stationary period and during convalescence.
3. Male, age 20; diagnosis typhoid; Ficker's reaction positive the third week.
4. Male, 23 years old; typhoid; reaction positive third week.
5. Girl of 17; typhoid; positive reaction the third week.
6. Boy, 17 years old; typhoid; reaction positive during stationary period.
7. Five-year-old child; typhoid; positive reaction.
8. Seventeen-year-old boy; typhoid; reaction positive during convalescence and in the following relapse.
9. Girl of 18; pulmonary phthisis, with high temperature; reaction negative.
10. Male, age 19; rheumatic polyarthritis and croupous pneumonia; no reaction.
11. A farmer, age 21; cerebrospinal meningitis; reaction negative.
12. A shoemaker, 52 years old; osteomyelitis; no reaction.
13. A 70-year-old woman; osteomyelitis; no reaction.

The author concludes, that in Ficker's reagent we possess a valuable and reliable typhoid diagnosticum, and on account of its practicability and simplicity is preferable to the original Gruber-Widal reaction.—*Medical Fortnightly.*

---

### TURPENTINE IN TYPHOID FEVER.

MOORE, (*Practitioner*, 1904, Vol. LXXVII., p. 138,) is a great believer in the value of turpentine in the treatment of typhoid fever. It acts as a diffusible stimulant, intestinal antiseptic, prevents chest complications and meteorism, and also has an action in checking hemorrhage. He prescribes it in the following combination:

| | | |
|---|---|---|
| ℞ | Spiritus terebinthinæ | ʒij. |
| | Spiritus ætheris nitrosi | ʒij. |
| | Spiritus chloroformi | ʒij. |
| | Emulsion amygdalæ ad | ℥vj. |

M.—Ft. mistura. Sig.—Shake the bottle. Half an ounce for a dose.

—*Therapeutic Review.*

---

IN CONSTIPATION AND OBESITY.

PROFESSOR W. S. BOGOSLOWSKY, (*British Medical Journal,*) from clinical observations on the action and value of a constant bitter water, draws the following conclusions (*Transactions* of the Moscow Section of the Society for the Preservation of Public Health, No. VI):

Systematic treatment with Apenta water is especially indicated for constipation produced by atony of the bowels, and it has the advantage that its use does not give rise to subsequent constipation.

Its action is more gentle than that of some other bitter waters because it contains less calcium sulphate and no magnesium chloride. It is probably owing to this circumstance that it does not cause crampy pains.

The efficiency of Apenta as a remedy for the systematic treatment of obesity is clinically established.

